# Ultrasound Radiation Force for the Assessment of Bone Fracture Healing in Children: An In Vivo Pilot Study

**DOI:** 10.3390/s19040955

**Published:** 2019-02-24

**Authors:** Siavash Ghavami, Adriana Gregory, Jeremy Webb, Mahdi Bayat, Max Denis, Viksit Kumar, Todd A. Milbrand, A. Noelle Larson, Mostafa Fatemi, Azra Alizad

**Affiliations:** 1 Department of Radiology, Mayo Clinic College of Medicine & Science, Rochester, MN 55905, USA; roudsari.seyed@mayo.edu (S.G.); gregory.adriana@mayo.edu (A.G.); webb.jeremy@mayo.edu (J.W.); max_f_denis@hotmail.com (M.D.); 2 Department of Physiology and Biomedical Engineering, Mayo Clinic College of Medicine & Science, Rochester, MN 55905, USA; mahdi.bayat@case.edu (M.B.); kumar.viksit@mayo.edu (V.K.); Milbrandt.Todd@mayo.edu (T.A.M.); fatemi.mostafa@mayo.edu (M.F.); 3 Department of Orthopedic Surgery, Mayo Clinic College of Medicine & Science, Rochester, MN 55905, USA; Larson.Noelle@mayo.edu

**Keywords:** ultrasound radiation force, bone fracture, bone healing, variational mode decomposition

## Abstract

Vibrational characteristics of bone are directly dependent on its physical properties. In this study, a vibrational method for bone evaluation is introduced. We propose a new type of quantitative vibro-acoustic method based on the acoustic radiation force of ultrasound for bone characterization in persons with fracture. Using this method, we excited the clavicle or ulna by an ultrasound radiation force pulse which induces vibrations in the bone, resulting in an acoustic wave that is measured by a hydrophone placed on the skin. The acoustic signals were used for wave velocity estimation based on a cross-correlation technique. To further separate different vibration characteristics, we adopted a variational mode decomposition technique to decompose the received signal into an ensemble of band-limited intrinsic mode functions, allowing analysis of the acoustic signals by their constitutive components. This prospective study included 15 patients: 12 with clavicle fractures and three with ulna fractures. Contralateral intact bones were used as controls. Statistical analysis demonstrated that fractured bones can be differentiated from intact ones with a detection probability of 80%. Additionally, we introduce a “healing factor” to quantify the bone healing progress which successfully tracked the progress of healing in 80% of the clavicle fractures in the study.

## 1. Introduction

Speed of fracture healing may vary from one individual to another and among different fracture locations based on anatomic location, fracture severity, patient physiological state, and treatment plan. Immobilization is the choice treatment for a majority of fractures. Determining the optimum duration of immobilization is important, as prolonged cast immobilization has multiple drawbacks, including muscle atrophy, joint stiffness, and skin breakdown. Furthermore, each cast change exposes the patient to the risk of cast saw burns or lacerations [1]. Paradoxically, excessive immobilization can delay the healing process, since bone formation needed for healing the fracture site is stimulated by mechanical stresses through mechanosensors within the osteocytes [2]. Significant debate exists amongst musculoskeletal care providers as to identifying when a fracture is healed sufficiently to discontinue immobilization and resume activities. Currently, the assessment of bone fracture healing is performed by serial radiographic and physical examinations [3]. Studies have shown the poor sensitivity of radiographic measurement in the prediction of healing in a rabbit model of bone fracture [4,5,6]. 

Conventional ultrasound in humans has been reported for detecting clavicle and forearm fractures with variable sensitivity [7]. A 1998 study [8] used conventional ultrasound to assess fracture healing in a preclinical canine model and found that a hyperechoic ultrasound signal correlated 100% of the time with the presence of hard fracture callus biopsy tissue. The development of quantitative tools for bone healing monitoring may assist physicians to identify the healing endpoint, thus preventing prolongation of the treatment course or incorrect timing of the removal of an external or internal fixation device. Several noninvasive quantitative techniques have been investigated to monitor bone fracture healing [9], including mechanical vibrational analysis [10,11,12] and acoustic emission [13,14]. Quantitative ultrasound (QUS) based on axial transmission has been proposed to assess normal and fractured bone [15,16].

The QUS technique involves placing a set of linearly arranged transducers (transmitters and receivers) along the bone axis. The receivers detect the ultrasound waves that have propagated along the cortical shell, and some of the generated parameters can be estimated for tissue characterization. Two-dimensional simulations on bone-mimicking plates, as well as in vivo studies, have investigated the potential of ultrasound axial transmission to evaluate fracture healing [15,16,17]. A model for fracture healing monitoring using QUS has been validated by experimental studies on ex vivo bovine femur and simulation studies [18]. Additionally, the effect of callus mineral composition has been reported by using simulation and experimental phantom studies [19]. Overall, both conventional ultrasound and QUS have variable results due to the presence of soft tissue around the bone. The impacts of overlying soft tissue on QUS and vibrational analysis in evaluating bone quality have been reported [20,21].

The utility of ultrasound radiation force (URF) for the characterization of soft tissues has been of interest for the past two decades. Vibro-acoustography, in particular, uses URF for imaging and tissue characterization [22,23]. The focused URF of vibro-acoustography induces vibrations in the object of interest. The frequency of those vibrations can be much smaller than that of the primary ultrasound used for URF. Object vibrations produce an acoustic field that is detected by a hydrophone sensitive to the frequency band of the induced vibrations. To construct an image of the object, the URF is scanned across it, usually in a raster fashion. The resulting image provides information on the object’s vibrational and mechanical characteristics. The temporal waveform of the URF depends on the particular application. Examples of URF waveforms include single frequency [22,24], multi-frequency [25], tone burst [26,27], and chirp signal [28]. Depending on the application, different types of transducers have been used to produce the URF, such as dual-element confocal [29], linear array [30], multi-row array [26], and sector array [31]. Vibro-acoustography has been used for imaging tissues and implants [32,33,34,35,36]. An overview of the application of this technique on various soft tissues has been previously reported [27]. Additionally, vibro-acoustography may be used for bone evaluation, as described in a recent study by Denis and colleagues [37]. The initial application of vibro-acoustography in the assessment of bone fracture and bone healing was presented in 2006 [38]. In that study, continuous URF was used to evaluate the resonance frequencies of ex-vivo animal bone. The premise behind their method was that bone fracture and the level of repair could be determined based on changes in the bone resonance frequency(ies). 

In this paper, we propose a quantitative URF method for detecting bone fracture and monitoring bone healing in human subjects. We applied focused URF excitations at four equi-distant points on the bone of interest. Resulting acoustic signals were used for wave arrival time and velocity estimation. The arrival time of the received signal at the hydrophone from each excitation included the effect of overlaying soft tissue. However, by measuring the time difference between the arrival times of the signals originating from two excitation points at known positions, it is possible to calculate the speed in bone in the vicinity of the excitation points. The signal measured by the hydrophone can be considered as a superposition of different modes or waves. Variational mode decomposition (VMD) [39] is an efficient method for decomposing a signal into an ensemble of band-limited intrinsic mode functions, and we used it to decompose the received signal into its constitutive modes and estimate the arrival time and velocity from the decomposed modes. Our proposed method successfully distinguished between fractured and normal bone with an 80% probability and was able to monitor the healing progress in 80% of the clavicular fractures.

## 2. Materials and Methods

### 2.1. Human Subjects

This prospective study has been conducted under an approved Mayo Clinic IRB (institutional review board) Protocol and is HIPPA (Health Insurance Portability and Accountability Act) Compliant. The study included 15 children with an obtained IRB approved informed consent/assent form, recruited between August 2016 and October 2017. Of the total, 12 had a single clavicle fracture and three had a single ulna fracture. Median age was 12 ± 4.1 years. There were 10 males (median age 11 ± 3.9 years) and five females (median age 8 ± 4.6). Signed written consent was obtained from the parents/guardians of each subject. Participants who could attend multiple visits post-fracture were considered for assessing bone healing and included 13 subjects with a clavicle fracture and three with an ulna fracture. The median times between the fracture date and the dates of the first, second, and third visits were 8 ± 2.5 days, 30 ± 8.7 days, and 45 ± 16.3 days, respectively. Clinical reports, including X-ray results and other clinical indicators reported by the physician, were used for the definitive assessment of fracture healing. 

### 2.2. Remote Acoustic Measurements Setup

**Figure 1:**
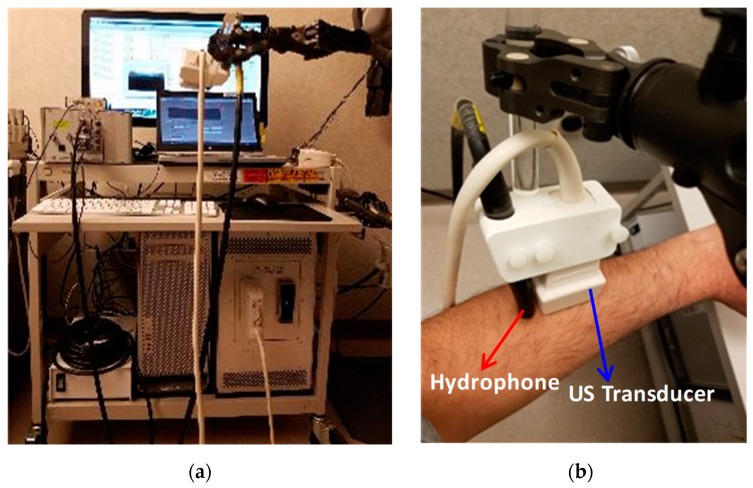
Experimental setup of URF bone measurement including: (**a**) Verasonics V-1 machine and probe-hydrophone assembly; (**b**) ultrasound transducer and hydrophone assembly placement on skin. Red arrow shows the hydrophone and blue arrow shows the ultrasound transducer.

A fully programmable ultrasound platform (Verasonics V-1, Kirkland, WA, USA) equipped with a linear array transducer (L7-4) was used to guide and generate the URF excitation. The URF signal consists of a five-cycle tone burst at a 5 MHz center frequency. The magnitude radiation force exerted by an acoustic plane wave in a 5 MHz center frequency transducer depends on the absorption coefficient and average acoustic intensity wave. The absorption coefficient directly depends on the frequency and the average acoustic intensity wave directly depends on the voltage of the transducer. By increasing the frequency, the absorption coefficient is increased, while the maximum tolerable voltage of each element in the transducer is decreased. We selected the frequency and voltage in order to achieve the maximum radiation force. Among the linear array transducers (Verasonics research system), L7-4 was the best candidate for our experimental setup. A Reson TC4034-1 hydrophone (Denmark) was used to detect the radiated acoustic pressure response. The safety measurements (i.e., temperature index and mechanical index) for the setup were performed; both were within the Food and Drug Administration limit. The hydrophone output was then filtered with an analogue eight-pole band-pass Bessel filter with a center frequency of 500 kHz, digitized at a sampling frequency of 100 Mega samples per second, and recorded on a computer. The transmitting linear array and the receiving hydrophone (RX) were placed in a fixture at a distance of 0.5 cm apart. Acoustic gel was used for coupling the hydrophone/probe to the skin. The experimental setup can be seen in Figure 1.

### 2.3. Problem Statement

We defined two hypothesis tests: one for distinguishing between intact and fractured bones and another for identifying the progression of the healing process in fractured bone. The first hypothesis test was defined based on the velocity of the wave received by the hydrophone. The second hypothesis test was defined based on the comparison of the wave velocity distribution in the fractured bone against that of the corresponding contralateral intact bone at different visits. 

In the first test, the velocity of the wave received by the hydrophone was considered as a decision variable for separating fractured from intact bones, which was defined as:

(1) $$\{\begin{array}{c} H_{F}:\;\;v<v_{THR}\\ H_{I}:\;\;v\geq v_{THR}, \end{array}$$

where $H_{F}$ and $H_{I}$ denote a set of fractured and intact bones, respectively; v is the wave velocity; and $v_{\mathrm{THR}}$ is the threshold of decision based on the wave velocity [9]. This hypothesis test was defined based on the fact that we would expect the wave velocity in intact bone to be higher than the wave velocity in fractured bone. To test the hypothesis in (1), we needed to estimate the wave velocity of the received signal. Hence, in the next Section 2.4.1 and Section 2.4.2, two methods are described for wave velocity estimation. For each subject, we measured the velocity of the fractured and the corresponding contralateral intact bone multiple times. Thus, for fracture detection, it was possible to compare the distributions of the velocity (or the corresponding time delay) in each pair of intact and fractured bones. 

The second hypothesis test was also based on comparing wave velocity distributions between fractured bone and the corresponding contralateral intact bone in each patient. To detect the progress of the healing process of each fractured bone over time, we compared the probability distribution function (PDF) of the estimated velocity in fractured bone to that of the contralateral intact bone at each visit using a rank sum test. The derived *p* value from the comparison of the pair of PDFs of velocity (or time delay) in patient l at visit m is denoted by $p_{l}\left(m\right)$. Thus, we defined a decision variable for detecting the progress of the healing process in each patient as follows:

(2) $$HD_{l}:\;=\{\begin{array}{cc} 1 & \forall m\in \left\{1,...,N_{l}\right\}:p_{l}\left(m+1\right)>p_{l}\left(m\right)\\ 0 & \exists m\in \left\{1,...,N_{l}\right\}:p_{l}\left(m+1\right)\leq p_{l}\left(m\right), \end{array}$$

where $N_{l}$ is the number of visits for patient l. If $p_{l}\left(m\right)$ is a monotonically increasing function in terms of visit m, we detect the healing process; otherwise, we cannot consider that sufficient healing progress has occurred in patient l. Moreover, if the *p* value in the first visit is less than 0.05, i.e., $p_{l}\left(1\right)<0.05$, and in the last visit, $p_{l}\left(N_{l}\right)$, is sufficiently large, the decision variable of $HD_{l}=1$ is considered as healing detection; otherwise, $HD_{l}=0$. The percentage of successful healing detection was defined by the quality healing factor (*QHF*) as:

(3) $$QHF=\frac{\sum_{l=1}^{L}HD_{l}}{L}\times 100,$$

where L is the number of patients. The advantage of comparing the PDF of velocities (or time delay) on different measurements of intact bone and bone with fracture as opposed to having only one statistic, such as the mean or median of velocity (or time delay), is that complete information on the PDF of velocity (or time delay) is maintained for comparing intact and fractured bone.

Based on these two hypothesis tests and the use of the rank sum test, we examined the performance of our method for detecting fractures and fracture healing in human subjects.

### 2.4. Acoustic Parameters Estimation

Parameters were estimated from the temporal characteristics of the acoustic response in order to capture changes in the bone. The wave velocity and arrival time differences of the acoustic waves are of particular interest for bone mechanical assessment. For simplicity of notations, we omitted the volunteer index over all the defined parameters; however, in the results section, the distribution of each defined parameter was compared for fractured and intact bones over groups of volunteers. The detailed methods for parameter estimation used for evaluating the performance of the hypothesis tests are presented in the subsections that follow.

#### 2.4.1. Wave Velocity Estimation

**Figure 2:**
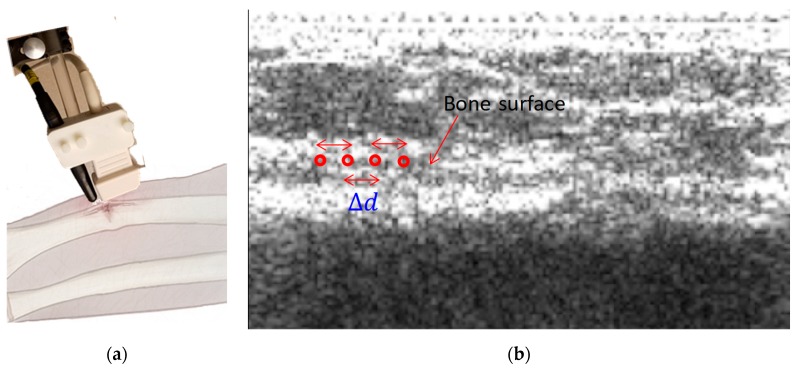
(**a**). Probe/hydrophone on the skin, where the fracture site is between the probe and hydrophone; (**b**) Conventional B-mode ultrasound-guided ultrasound with four radiation force excitation beam focuses (red circles) Δd apart. Time delay measurements are conducted between two excitations. The hydrophone is located at the left side of the image. The arrow shows the bone surface.

The velocity of the acoustic wave was estimated from the arrival time difference measurements along the bone at each visit. The probe/hydrophone assembly was positioned along the bone, such that an imaginary line between the probe and the hydrophone was parallel to the long axis of the bone. In the case of fractured bone, the fracture position was marked on the skin by the clinical staff. Using this mark as the guide, the probe/hydrophone assembly was placed over the fracture, such that the focus of the URF was on one side of the fracture while the hydrophone was on the other side of the fracture, as shown in Figure 2a. To make the measurements on the contralateral intact bone, the probe/hydrophone assembly was placed at an approximately similar position.

Multiple measurements were performed by focusing the URF at different positions on the bone. The distance between the URF focus (TX) and the receiving hydrophone (RX) is $d_{i}=d_{0}+i\Delta d\;\mathrm{cm}$, where $d_{0}=1.2\;\mathrm{cm}$, i∈{0,1,2,3}, and Δd=1.2 mm (Figure 2b). For the measured signal at distance $d_{i}$, the kth measurement of mth patient visit is denoted by $x_{i}^{\left(k,m\right)}\left(nT_{s}\right)$, where $T_{s}=1/F_{s}$ is the sampling interval and $F_{s}$ is the sampling frequency, as follows:

$$x_{i}^{\left(k,m\right)}:=[x_{i}^{\left(k,m\right)}\left[1\right],...,x_{i}^{\left(k,m\right)}\left[N\right]],$$

where $x_{i}^{\left(k,m\right)}\left[n\right]=x_{i}^{\left(k,m\right)}\left(nT_{s}\right)$ is the $n^{\mathrm{th}}$ sample at distance $d_{i}$ and $k^{\mathrm{th}}$ measurement of $m^{\mathrm{th}}$ patient visit, 1≤k≤K, 1≤n≤N, 1≤m≤M. The variables K, N, and M are the number of measurements at each patient visit, the number of samples in each measurement, and the number of patient visits, respectively. We measured at K different sites on the bone. At all sites, the fracture was between the excitation point of the probe and the hydrophone.

By cross-correlating measured signals of $x_{i}^{\left(k,m\right)}$ and $x_{j}^{\left(k,m\right)}$, where i,j∈{0,1,2,3} and i≠j, the bone wave velocity of $k^{\mathrm{th}}$ measurement and $m^{th}$ patient visit was estimated as follows:

(4) $$v_{ij}^{\left(k,m\right)}:\;=\frac{\left(j-i\right)\Delta d}{\Delta \tau_{ij}^{\left(k,m\right)}},$$

where $\Delta \tau_{ij}^{\left(k,m\right)}$ is the time delay between the signals of $x_{i}^{\left(k,m\right)}$ and $x_{j}^{\left(k,m\right)}$, which was derived from the following optimization problem:

(5) $$\begin{array}{l} \Delta \tau_{ij}^{\left(k,m\right)}:\;=T_{s}\arg\max{\hat{R}}_{ij}^{\left(k,m\right)}\left(\Delta n\right)\\ s.t.\;\;\frac{\left(j-i\right)\Delta d}{v_{\max}}\leq \Delta \tau_{ij}^{\left(k\right)}\leq \frac{\left(j-i\right)\Delta d}{v_{\min}} \end{array}$$

where $v_{\max}$ and $v_{\min}$ are, respectively, the maximum and minimum values of the allowed velocity in the propagation medium (i.e., bone); and ${\hat{R}}_{ij}^{\left(k\right)}\left(\Delta n\right)$ is the estimated weighted cross correlation between $x_{i}^{\left(k,m\right)}$ and $x_{j}^{\left(k,m\right)}$, which was obtained by:

(6) $${\hat{R}}_{ij}^{\left(k,m\right)}\left(\Delta n\right)=\sum_{n=1}^{N_{C}}x_{i}^{\left(k,m\right)}\left[n\right]w\left[n\right]x_{j}^{\left(k,m\right)}\left[n+\Delta n\right]w\left[n+\Delta n\right],$$

where i,j∈{0,1,2,3}, j<i, and $N_{C}$ are the number of samples used for the estimation of the cross correlation. Also, $w\left[n\right]:=w\left(nT_{s}\right)$ denotes the Tukey window (tapered cosine) in the time domain and was defined as:

(7) $$w\left(t\right):\;=\{\begin{array}{ll} \frac{1}{2}\left(1+\cos\left(\frac{2\pi }{r}\left(t-\frac{r}{2}\right)\right)\right), & 0\leq t<\frac{r}{2}\\ 1, & \frac{r}{2}\leq t<1-\frac{r}{2}\\ \frac{1}{2}\left(1+\cos\left(\frac{2\pi }{r}\left(t-1+\frac{r}{2}\right)\right)\right), & 1-\frac{r}{2}\leq t\leq 1, \end{array}$$

where 0≤r≤1 is the roll-off factor. To compare the bones with and without fracture, we estimated the sample mean, sample median, and sample standard deviation of the estimated time delay and bone wave velocity for each measurement and each volunteer. The mean, median, and standard deviation of $\Delta \tau_{ij}^{\left(k,m\right)}$ at mth patient visit were derived as follows:

(8a) $$\Delta {\bar{\tau }}^{\left(m\right)}:\;=\frac{1}{6K}\sum_{k=1}^{K}\sum_{j=i}^{3}\sum_{i=0}^{3}\Delta \tau_{ij}^{\left(k,m\right)}$$

(8b) $$\Delta {\tilde{\tau }}^{\left(m\right)}:\;=median\left(\Delta \tau_{ij}^{\left(k,m\right)}\right)$$

(8c) $$\sigma_{\Delta \tau }^{\left(m\right)}:\;=\frac{1}{6K-1}\sum_{k=1}^{K}\sum_{j=i}^{3}\sum_{i=0}^{3}{\left(\Delta \tau_{ij}^{\left(k,m\right)}-\Delta {\bar{\tau }}^{\left(m\right)}\right)}^{2}.$$

In a similar way the mean, median, and standard deviation of $v_{ij}^{\left(k,m\right)}$ at mth patient visit were obtained by:

(9a) $${\bar{v}}^{\left(m\right)}:\;=\frac{1}{6K}\sum_{k=1}^{K}\sum_{j=i}^{3}\sum_{i=0}^{3}v_{ij}^{\left(k,m\right)}$$

(9b) $${\tilde{v}}^{\left(m\right)}:\;=median\left(v_{ij}^{\left(k,m\right)}\right)$$

(9c) $$\sigma_{v}^{\left(m\right)}:\;=\frac{1}{6-1}\sum_{k=1}^{K}\sum_{j=i}^{3}\sum_{i=0}^{3}\left(v_{ij}^{\left(k,m\right)}-\bar{v}\right).$$

#### 2.4.2. Variational Mode Decomposition

The signal received by the hydrophone may be considered as a superposition of different components. The measured signal was decomposed into a discrete number of sub-signals (modes), $u_{p,i}^{\left(k,m\right)}$, which had specific sparsity properties related to the bandwidth of the mode. These modes are reproducing the measured signal. The bandwidth of $u_{p,i}^{\left(k,m\right)}$, using a single side-band frequency spectrum, was derived for $u_{p,i}^{\left(k,m\right)}$ from the corresponding analytic signal with a Hilbert transform. By multiplying an exponential with the estimated center frequency, the mode’s frequency spectrum was demodulated and transferred to the baseband. Bandwidth was estimated using a squared $L_{2}$-norm of the gradient. Therefore, the resulting constrained variational problem for $x_{i}^{\left(k,m\right)}\left(t\right)$ was obtained by minimizing the sum of the gradients of the Hilbert transform of the base band modes [39] as follows:

(10) $$\begin{array}{l} \underset{\left\{u_{p,i}^{\left(k,m\right)}\right\},\left\{\omega_{p,i}^{\left(k,m\right)}\right\}}{\min}\left\{\sum_{p=1}^{P}{\left\|\frac{\partial }{\partial t}\left[\left(\delta \left(t\right)+\frac{j}{\pi t}\right)*u_{p,i}^{\left(k,m\right)}\left(t\right)\right]e^{-j\omega_{p}t}\right\|}_{2}^{2}\right\}\\ s.t.\;\;\sum_{p=1}^{P}u_{p,i}^{\left(k,m\right)}=x_{i}^{\left(k,m\right)}\left(t\right), \end{array}$$

where $\left\{u_{p,i}^{\left(k,m\right)}\right\}=\left\{u_{1,i}^{\left(k,m\right)},...,u_{P,i}^{\left(k,m\right)}\right\}$ is the set of all modes; $\left\{\omega_{p,i}^{\left(k,m\right)}\right\}=\left\{\omega_{1,i}^{\left(k,m\right)},...,\omega_{P,i}^{\left(k,m\right)}\right\}$ is the set of center frequencies; P is the number of modes; δ(t) is the Dirac delta function; and ∗ and ${\left\|\right\|}_{2}^{2}$ denote the convolution operation and $L_{2}$ norm, respectively. The augmented Lagrangian function for the optimization of (10) was obtained by:

(11) $$\begin{array}{l} L\left(\left\{u_{p,i}^{\left(k,m\right)}\right\},\left\{\omega_{p,i}^{\left(k,m\right)}\right\},\lambda_{i}^{\left(k,m\right)}\right):=\alpha \sum_{p=1}^{P}{\left\|\frac{\partial }{\partial t}\left(\delta \left(t\right)+\frac{j}{\pi t}\right)*u_{p,i}^{\left(k,m\right)}\left(t\right)e^{-j\omega_{p}t}\right\|}_{2}^{2}+\\ {\left\|x_{i}^{\left(k,m\right)}\left(t\right)-\sum_{p=1}^{P}u_{p,i}^{\left(k,m\right)}\left(t\right)\right\|}_{2}^{2}+<\lambda_{i}^{\left(k,m\right)}\left(t\right),x_{i}^{\left(k,m\right)}\left(t\right)-\sum_{p=1}^{P}u_{p,i}^{\left(k,m\right)}\left(t\right)> \end{array}$$

where α is the weight of the bandwidth constraint and $\lambda_{i}^{\left(k,m\right)}\left(t\right)$ is the Lagrange multiplier. The solution of the original minimization problem was a saddle point of the augmented Lagrangian in a sequence of iterative sub-optimizations called the alternate direction method of multipliers [40] (see the Appendix for more details about the steps of optimization in VMD). 

Therefore, by estimating modes of the measured signal using the optimization algorithm of VMD for $x_{i}^{\left(k,m\right)}$, we selected two modes for velocity estimation: the first mode with the minimum center frequency, $u_{1,i}^{\left(k,m\right)}$, and the mode with the maximum power, $u_{\max,i}^{\left(k,m\right)}=\underset{p}{\max}\left|u_{p,i}^{\left(k,m\right)}\left(t\right)\right|$. The arrival times difference and velocity estimation were derived, respectively, from (5) and (4) by replacing $x_{i}^{\left(k,m\right)}$ with $u_{1,i}^{\left(k,m\right)}$ or $u_{\max,i}^{\left(k,m\right)}$ in (6).

#### 2.4.3. Accuracy of Time Delay Estimation

To assess the accuracy of the time delay, Δτ, estimation in intact bones as the control group, we estimated the coefficient of variation (CV) of Δτ for intact bone over all measurement sites and participant visits with different method of time delay estimation, denoted by $CV_{total}\left(\Delta \tau \right)$, which was defined as:

(12) $$CV_{total}:\;=\frac{\sigma_{total}}{\mu_{total}},$$

where

(13a) $$\mu_{total}:\;=\frac{1}{6KM}\sum_{m=1}^{M}\sum_{k=1}^{K}\sum_{j=i}^{3}\sum_{i=0}^{3}\Delta \tau_{ij}^{\left(k,m\right)}$$

(13b) $$\sigma_{total}:\;=\frac{1}{6KM-1}{\sum_{m=1}^{M}\sum_{k=1}^{K}\sum_{j=i}^{3}\sum_{i=0}^{3}\left(\Delta \tau_{ij}^{\left(k,m\right)}-\mu_{total}\right)}^{2}.$$

Also, we compared CV for mth patient visit as follows:

(14) $$CV^{\left(m\right)}:\;=\frac{\sigma_{\Delta \tau }^{\left(m\right)}}{\Delta {\bar{\tau }}^{\left(m\right)}},$$

where

(15a) $$\Delta {\bar{\tau }}^{\left(m\right)}:\;=\frac{1}{6K}\sum_{k=1}^{K}\sum_{j=i}^{3}\sum_{i=0}^{3}\Delta \tau_{ij}^{\left(k,m\right)}$$

(15b) $$\sigma_{\Delta \tau }^{\left(m\right)}:\;=\frac{1}{6K-1}\sum_{k=1}^{K}\sum_{j=i}^{3}\sum_{i=0}^{3}{\left(\Delta \tau_{ij}^{\left(k,m\right)}-\Delta {\bar{\tau }}^{\left(m\right)}\right)}^{2}.$$

To assess the accuracy of $\Delta {\bar{\tau }}^{\left(m\right)}$ over different patient visits, we calculated the coefficient of variation of $\Delta {\bar{\tau }}^{\left(m\right)}$, denoted by $CV_{mean}\left(\Delta \tau \right)$, which was defined as:

(16) $$CV_{mean}:\;=\frac{\sigma_{mean}}{\mu_{mean}},$$

where

(17a) $$\mu_{mean}:\;=\frac{1}{M}\sum_{m=1}^{M}\Delta {\bar{\tau }}^{\left(m\right)}$$

(17b) $$\sigma_{mean}:\;=\frac{1}{M-1}\sum_{m=1}^{M}{\left(\Delta {\bar{\tau }}^{\left(m\right)}-\mu_{mean}\right)}^{2}.$$

## 3. Results

**Figure 3:**
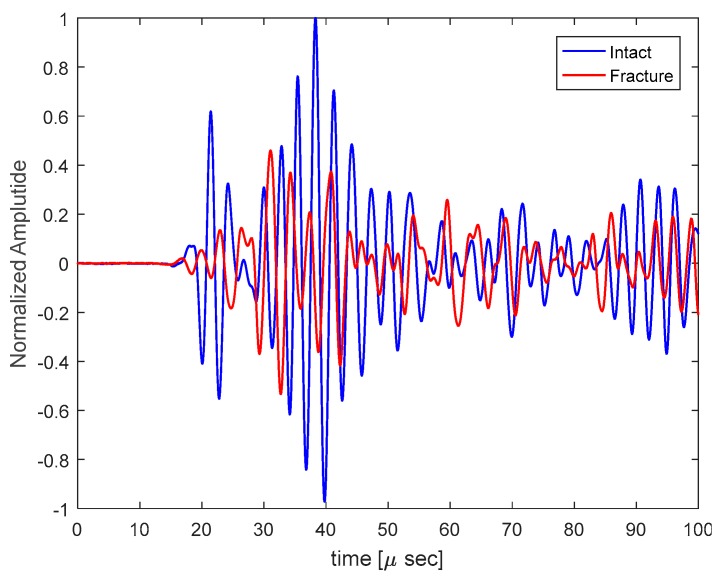
A typical time domain signal received by the hydrophone from intact clavicle bone and clavicle bone with fracture; the signal starting point is 14 µs.

The vibro-acoustic measurements for the fractured and intact bones demonstrated significant differences in velocity and arrival times. The number of measurements, K, was equal to three in each bone. The roll-off factor of the Tukey window in the estimation of velocity and time delay, r, was 0.25. The processing window duration, $T_{W}$, was considered to be 25 µs to analyze only the first part of the received signal by the hydrophone, which mainly includes the bone component wave because the wave propagates faster in bone than in soft tissue. In this section, $v_{\max}=4080\;m/s$ and $v_{\min}=1000\;m/s$ are considered for solving the optimization problem in (5); $v_{\max}$ is obtained from the maximum velocity of the wave in the bone, and $v_{\min}$ is obtained from a lower bound for the minimum velocity of the wave in soft tissue. Typical signals received by the hydrophone from intact and fractured bones are shown in Figure 3.

**Figure 4:**
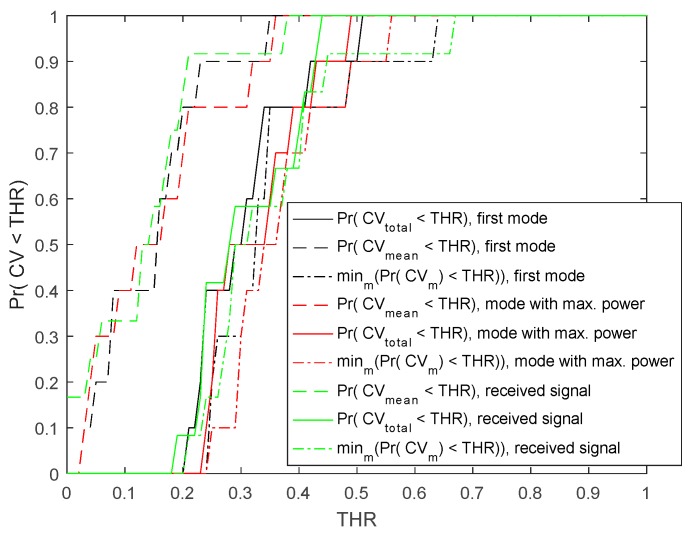
$\mathrm{Pr}\{CV_{total}\leq THR\}$, $\mathrm{Pr}\{CV_{mean}\leq THR\}$, and $\underset{m}{\min}\mathrm{Pr}\{CV_{m}\leq THR\}$ for the first mode, mode with maximum power, and received signal for the clavicle bone with completed visit, respectively.

Figure 4 depicts $\mathrm{Pr}\{CV_{total}\leq THR\}$, $\mathrm{Pr}\{CV_{mean}\leq THR\}$, and $\underset{m}{\min}\mathrm{Pr}\{CV_{m}\leq THR\}$ for the first mode, which is the mode with the minimum center frequency, the mode with the maximum power, and the received signal for participants with clavicle fracture who completed at least three visits, respectively. Here, Pr{ } denotes the probability distribution function and THR is a threshold value. As shown by the example in the figure, when we used the first mode of VMD for time delay estimation, $\mathrm{Pr}\left\{CV_{mean}\leq 0.2\right\}=0.8$, $\mathrm{Pr}\left\{CV_{total}\leq 0.34\right\}=0.8$, and $\underset{m}{\min}\mathrm{Pr}\{CV^{\left(m\right)}\leq 0.35\}=0.8$, indicating that the accuracy of the Δτ estimation was fairly similar across our normal bone population.

**Figure 5:**
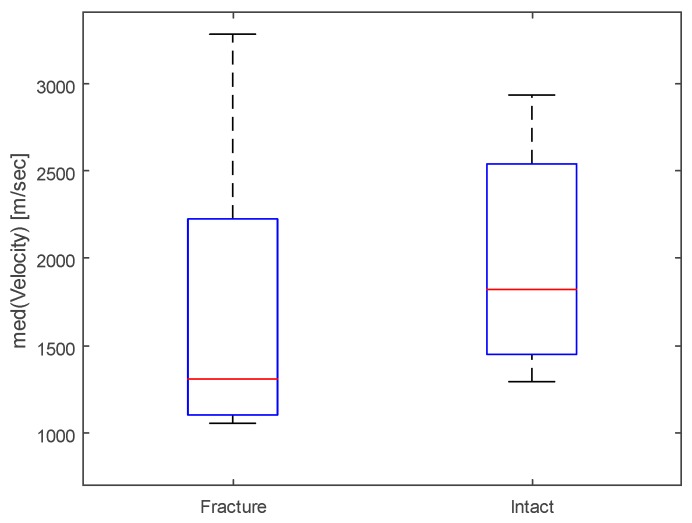
Box-Whisker plots of the median of estimated bone velocity in fractured clavicles versus normal clavicles, *p* < 0.05.

The median of the estimated bone velocity in fractured versus normal clavicles, using ${\tilde{v}}^{\left(m\right)}$, is plotted for comparison in Figure 5. Velocities significantly differed between groups (*p* < 0.05) using the rank sum test. 

Performance of fracture detection based on a pair-wise comparison between intact and fractured bone for each patient is listed in Table 1. The best performance of fracture detection was 0.80 using the first mode of VMD as the input of the cross correlation technique.

Table 2 shows the estimated QHF based on the time delay and velocity for different estimation methods: constrained velocity and first mode of VMD. We found that the healing process could be detected in 80% of fractured clavicle bones with this method. 

## 4. Discussion

Detection of the fracture and healing process of bone is critical. In this paper, a new noninvasive and quantitative URF method was proposed for the detection of bone fracture and healing. We found that waves generated by ultrasound radiation force excitation vary in speed, depending on the healing of the broken bone to which they are applied. In particular, the velocity and arrival time of the generated waves differed significantly between intact and fractured bone. Using our method, the statistical analysis revealed an 80% detection rate in bones with fracture. In monitoring the healing process, our method, based on an estimation of the time delay, demonstrated a detection rate of 80% in the clavicle bones and around 70% of both clavicle and ulna bones. Conventional ultrasound has also been reported for the detection of clavicle and forearm fractures, with variable sensitivity [7]. The development of quantitative tools for bone healing monitoring may assist physicians in identifying the healing endpoint, thus preventing prolongation of the treatment course or incorrect timing of fixation device removal. Several noninvasive, quantitative techniques to monitor bone fracture healing have been investigated [9,10,11,12,13,14,15,16,18,19]. These studies have shown a correlation between the ultrasound wave velocity and health status of bone, therefore proposing that the ultrasound wave is an accurate method for assessing fracture. 

To assess reliability in the estimation of time delay and velocity in normal bone, the coefficient of variation of time delay and velocity was calculated in the normal bone population. All measurements showed that the value was almost 20%, indicating a reliable estimation of time delay and velocity. 

There are some limitations to this work. First, the bone was supposed to be an isotropic material. Consequently, the elastic coefficient was assumed to be uniform in all directions. Second, the receiving hydrophone is omnidirectional, as opposed to a directionally focused transducer. Consequently, the received pressure has contributions in all directions; however, the hydrophone is held perpendicular to the bone surface so that the displacement toward the bone (deflection) will be the main contributor to the received pressure. Third, changes in the wave velocity with respect to bone density were not investigated. Velocity is not only dependent on the elastic modulus, but is also linked to density. Fourth, using VMD, we decomposed the signal received by the hydrophone to multiple modes and analyzed the mode with the maximum power for velocity estimation. The decomposed modes, though, could have been different from the waves propagating in the bone tissue material. The results of simulation and experimental ex vivo studies on cortical bone for analyzing the lamb modes using guided waves have also been reported [41,42,43,44].

Further investigation is required to improve the classification of fractured and normal bone and to be able to adequately monitor the healing process. Additional acoustic parameters, such as attenuation, may further enhance the accuracy of classification. A larger dataset can aid in a more accurate performance in identifying fractures and detecting bone healing.

## 5. Conclusions

Waves generated by ultrasound radiation force excitation of intact bone and bone with fracture demonstrated a significant difference in the velocity and arrival time, and in the clavicle in particular. The healing process of bone with fracture was also detected by identifying the wave velocity in fractured bone over time and comparing it to corresponding intact bone. Results indicated that waves generated by ultrasound radiation force excitation vary in velocity, depending on the health of the bone to which they are applied. 

## Figures and Tables

**Table 1:** Performance of fracture detection based on a pair-wise comparison between intact and fractured bone.

Input of Delay Estimation Algorithm	Decision Variable	Threshold on *p* Value	
		0.05	0.1
Constrained velocity	Time delay	40%	60%
	Velocity	73%	73%
First mode	Time delay	80%	80%
	Velocity	67%	80%

**Table 2:** Estimated quality of healing factor (QHF) based on time delay and velocity for different estimation methods: received signal and first mode of variational mode decomposition (VMD).

Estimation Method	Bone Type	Decision Variable	QHF
Constrained velocity	Clavicle	Time delay	60%
		Velocity	60%
	Clavicle + Ulna	Time delay	54%
		Velocity	62%
First mode of VMD	Clavicle	Time delay	80%
		Velocity	70%
	Clavicle + Ulna	Time delay	69%
		Velocity	62%

## Appendix A

The algorithm used for optimization in VMD is summarized below. Here, $\hat{•}$ denotes the Fourier transform of •. In this algorithm, for simplicity of notation, superscript (k,m) and the subscript of i are omitted, hence ${\hat{x}}_{i}^{\left(k,m\right)}\left(\omega \right)$ and ${\hat{u}}_{i,p}^{\left(k,m\right)}\left(\omega \right)$ are denoted by $\hat{x}\left(\omega \right)$ and ${\hat{u}}_{p}\left(\omega \right)$, respectively.

**Algorithm A1.** Steps of optimization for variational mode decomposition.
$\begin{array}{l} \mathrm{Initilize}\;\left\{{\hat{u}}_{p}^{1}\right\},\left\{\omega_{p}^{1}\right\},{\hat{\lambda }}^{1},n\leftarrow 0\\ \mathrm{repeat} \end{array}$ $\begin{array}{l} n\leftarrow n+1\\ \mathrm{for}\;p=1:P\;\mathrm{do}\\ \;\mathrm{Update}\;{\hat{u}}_{p}\;\mathrm{for}\;\mathrm{all}\;\omega \geq 0: \end{array}$ ${\hat{u}}_{p}^{n+1}\left(\omega \right)\leftarrow \frac{\hat{x}\left(\omega \right)-\sum_{i<p}{\hat{u}}_{i}^{n+1}\left(\omega \right)-\sum_{i>p}{\hat{u}}_{i}^{n}\left(\omega \right)+\frac{{\hat{\lambda }}^{n}\left(\omega \right)}{2}}{1+2\alpha {\left(\omega -\omega_{p}^{n}\right)}^{2}}$ $\begin{array}{l} \mathrm{Update}\;\omega_{p}:\\ \;\omega_{p}^{n+1}\leftarrow \frac{\int_{0}^{\infty }\omega {\left|{\hat{u}}_{p}^{n+1}\left(\omega \right)\right|}^{2}d\omega }{\int_{0}^{\infty }{\left|{\hat{u}}_{p}^{n+1}\left(\omega \right)\right|}^{2}d\omega } \end{array}$ $\begin{array}{l} \mathrm{end}\;\mathrm{for}\\ \mathrm{Dual}\;\mathrm{ascent}\;\mathrm{for}\;\mathrm{all}\;\omega \geq 0:\\ {\hat{\lambda }}^{n+1}\left(\omega \right)\leftarrow {\hat{\lambda }}^{n}\left(\omega \right)+\tau \left(\hat{x}\left(\omega \right)-\sum_{p}{\hat{u}}_{p}^{n+1}\left(\omega \right)\right) \end{array}$ $\mathrm{until}\;\mathrm{convergence}:\sum_{p=1}^{P}{{\left\|{\hat{u}}_{p}^{n+1}-{\hat{u}}_{p}^{n}\right\|}_{2}^{2}/\left\|{\hat{u}}_{p}^{}\right\|}_{2}^{2}<\varepsilon$
